# Oil Sorption Properties of Centrifugally Spun Polyisobutylene-Based Thermoplastic Elastomer Microfibers

**DOI:** 10.3390/polym16182624

**Published:** 2024-09-17

**Authors:** József Kántor, Gusztáv Fekete, Attila Levente Gergely

**Affiliations:** 1 Department of Mechanical Engineering, Faculty of Technical and Human Sciences, Sapientia Hungarian University of Transylvania, Târgu Mureş, Târgu Mureş/Corunca, Calea Sighișoarei nr. 2., 540485 Târgu Mureş, Romania; kantorjozsef@ms.sapientia.ro; 2 Department of Material Science and Technology, Audi Hungária Faculty of Vehicle Engineering, Széchenyi István University, Egyetem tér 1, 9026 Győr, Hungary

**Keywords:** fibers, centrifugal spinning, oil sorption, polyisobutylene, thermoplastic elastomer

## Abstract

Fiber-based sorbent materials are an essential part of containing oil spills, thus preventing ecological damage. Poly(styrene-*b*-isobutylene-*b*-styrene) thermoplastic elastomer fibers were successfully produced by centrifugal spinning. Scanning electron microscopy revealed that the fibers were bead free and smooth-surfaced, with an average fiber diameter of 5.9 ± 2.3 μm. Contact angle measurements proved the highly hydrophobic (water contact angle of 126.8 ± 6.4°) and highly oleophilic nature of the fiber mat. The sorption and retention capacities of the fiber mat were tested for various oils and benchmarked against polypropylene as the industry standard and polystyrene, which is widely used in the literature. The oil uptake of the fiber mat showed a strong correlation with the viscosity of the oil, resulting in sorption capacities of 10.1 ± 0.8 g/g for sunflower oil, 19.9 ± 2.1 g/g for motor oil, and 23.8 ± 1.8 g/g for gear oil. Oil–water separation tests were also conducted, resulting in ~100% oil removal. The thermoplastic elastomer fiber mat outperformed the industry standard; however, the polystyrene fiber mat demonstrated the best oil sorption performance.

## 1. Introduction

Oil spills, whether they happen on land or sea, have a detrimental effect on the environment [1,2]; however, larger oil spills usually occur in marine ecosystems. There are four techniques to mediate oil cleanup from the marine environment: chemical (dispersant and emulsion breakers), in situ burning, mechanical (skimmers and sorbents), and bioremediation [3,4]. All methods have their advantages and disadvantages and can be justified in their use; however, mechanical methods do not involve the use of added chemicals in situ or the generation of toxic gasses by burning the oil. Furthermore, they can can be executed on a timescale that can prevent ecological damage, unlike in the case of bioremediation.

Sorbents, which are described as porous structures that can collect and retain liquids, play an important role in oil–water separation in the framework of mechanical cleanup methods [5]. The most important properties of sorbents in oil collection applications are their hydrophobicity and oleophilicity. Sorbents are classified into four different categories by the ASTM F726 standard [6]: type I sorbents are the ones for which thickness is much smaller than the length and width (films, pads, and sheets), type II sorbents have loose structures and are made of unconsolidated particulate material that is handled with scoops or similar tools since it has no form or strength to be handled otherwise, type III uses a permeable fabric or netting to confine the sorbent material into a predetermined shape to form a pillow or a boom, and type IV sorbents are named agglomeration units consisting of strands, fibers, open netting, or other forms that allow viscous oil to penetrate into the structure [6].

Fibrous structures consisting of fibers with dimensions on the nano- and micrometer scales are an excellent choice in oil–water separation applications due to their high surface-to-volume ratio. In particular, polymeric fibers are great candidates to be used as sorbents since the hydrophobicity and oleophilicity of the fibers can be changed by the employment of different polymeric materials or with the use of surface treatments [5]. There are several ways to produce polymeric fibers; however, the most widely adopted method by researchers is single needle electrospinning. Electrospinning requires a simple setup [7], which is easy to use and it produces fibers on the nanometer scale [8]. The process has been described in great detail in the literature [8,9]; however, there are several shortcomings that prevent its wide industrial application: a high electric field is required (500–1000 V/cm), the solution used has to be electrically conductive, and the production rate is very low, 0.01–0.1 g/h dry fiber [10,11]. The production rate can be increased by the use of needleless electrospinning, 40 g/h has been reported [12]; however, the required equipment becomes more complicated and expensive, hindering the widespread application of this method. Centrifugal spinning is an emerging technique for fiber generation that promises a high production rate, while the polymeric solution used does not have to be electrically conductive [13,14,15]. The process requires a chamber, filled with the polymer solution, to be spun at high rotational speed (4000–15,000 rpm), and the polymeric solution bursts through the capillaries located on the side of chamber, forming polymer solution jets. The jets are elongated by the occurring centrifugal forces, the solvent evaporates, and the dry fibers deposit on a collector. Fibers with diameters ranging from 500–600 nm to a couple of μm have been produced by centrifugal spinning at production rates of 15–75 g/h dry fiber [13,14,15]. The aforementioned techniques use polymer solutions to produce fibers; however, fibers can be produced from polymer melts as well. In particular, melt blowing is used to produce fibers out of polymers that are more difficult to dissolve (such as polypropylene). This method requires the use of a high-velocity air stream applied to the molten polymer to produce fibers with diameters ranging from 0.3 to 10 μm [16,17,18]. While melt blowing eliminates the use of solvents to produce fibers, it requires more energy to melt the polymer material. Furthermore, heat-sensitive polymers or additives cannot be processed, and there is less control of fiber dimension [16].

Centrifugal spinning has barely been used in the literature to prepare fiber mats to be used in oil collection applications. Polystyrene (PS) fibers were prepared by Doan et al. with the use of centrifugal spinning from a 22% *w*/*w* solution with a solvent system of tetrahydrofuran (THF) and dimethylformamide (DMF). The resulting fibers had an average fiber size of 4–6 μm, produced at 15,000 rpm and 100 mL/h volumetric flow rate. They found a profound correlation between the porosity of the fibers and the solvent system composition. The water contact angle (WCA) was 145° and the oil uptake was 30–50 g/g depending on the fiber porosity [19]. Zhang et al. prepared polylactic acid fibers with the aid of centrifugal spinning from a 6–8% *w*/*w* solution, resulting in 4 μm thick fibers. The fiber mat had a WCA of 116°, and the oil collection capacity was 25–30 g/g [20].

Synthetic polymer materials have great potential as sorbents since they provide a wide variety of chemical structures that can be matched to the chemical structure of the collected oil. For instance, PS can be used for aromatic oils, while polyethylene (PE), polypropylene (PP), or polyisobutylene (PIB) can be used for aliphatics. PP fibers (3–7 μm) have been processed by melt electrospinning, showing an oil sorption capacity of 80–129 g/g [21], whereas porous crosslinked butyl rubber sheets showed an oil sorption capacity of 23 g/g for crude oil [22].

Poly(styrene-b-isobutylene-b-styrene) triblock copolymer (SIBS) is a thermoplastic elastomer that contains both aromatic and aliphatic structures, due to the presence of styrene and isobutylene repeat units, respectively. SIBS is amorphous, with great biostability and fatigue properties. SIBS, being a thermoplastic elastomer that is physically crosslinked, allows for the recycling of such material at elevated temperatures, in contrast to crosslinked rubber. Also, the elastic nature of SIBS provides greater flexibility when compared to thermoplastics, such as PS, PP, or PE [23]. SIBS retained ~100% of its tensile strength after being exposed to boiling 65% nitric acid for 30 min, suggesting a high acid resistance [23]. In another study, SIBS suffered no loss of molar mass due to hydrolysis in either acidic (pH 2) or alkaline (pH 12) media at 70 °C after 7 days of exposure [24]. Fiber production using SIBS by electrospinning has been rather limited due to the non-polar nature of the resulting polymer solution. Additives had to be added to the polymer solution to increase the conductivity, such as polypyrrole and p-toluenesulfonate hexahydrate [10], or polyethylene glycol [11]. Despite these results, Lim et al. managed to prepare SIBS fibers with 0.3–2 μm diameter using the THF and toluene solvent system. The fiber mat showed a near-superhydrophobic character with a WCA of 146° [25].

In the past five years, published research about fibrous sorbent devices has featured fewer studies with petroleum-based synthetic polymers in favor of biodegradable synthetic polymers, such as polylactic acid (PLA) [20], or natural fibers like cotton, wool, kapok, and cellulose. The measured oil sorption for these fibers ranges from 5–40 g/g in their unaltered state up to 85 g/g when surface modification is applied [26,27,28,29].

We previously reported the fabrication of SIBS fibers by centrifugal spinning [30]. We found that the polymer solution concentration, the rotational speed, and the solvent system composition used have a profound effect of the spinnability of SIBS. The optimal centrifugal spinning conditions were determined to be 25% *w*/*w* concentration in an 80:20 (*w*/*w*) THF–toluene solvent system with 8000 rpm rotational speed and 60 mL/h flow rate. The resulting fibers we bead free and smooth-surfaced, with an average diameter of 3.68 µm.

The aim of this work was to test, for the first time, the oil sorption capability of SIBS thermoplastic elastomer fiber mats produced by centrifugal spinning. SIBS, due to its chemical structure, contains both aromatic and aliphatic phases, thus holding high potential to be used as a sorbent for a wide range of oils. Furthermore, the oil sorption and retention capabilities were investigated as a function of oil viscosity and type. The results were benchmarked against PP as the industry standard and PS, which is widely used in the literature in oil sorption applications.

## 2. Materials and Methods

### 2.1. Materials

Poly(styrene-*b*-isobutylene-*b*-styrene) (SIBS; Sibstar 073T, M_n_ = 65,000 g/mol, 70/30 *w*/*w* PIB/PS; Kaneka Corporation, Tokyo, Japan), polystyrene (PS; MFI = 10.9 g/10 min at 200 °C, 5 kg load, and 8 min preheat time, M_n_ = 110,000 g/mol; donated by Viessmann Transilvania SRL, Târgu Mureş, Romania), tetrahydrofuran (THF; reagent grade; VWR), dimethylformamide (DMF; reagent grade; VWR), toluene (reagent grade; VWR), sunflower oil (food grade), 5W30 motor oil (Castrol Edge), 85W140 mineral transmission gear oil (Kross Trans GL-5), and standard diesel fuel (MOL Romania Petroleum Products SRL, Bucharest, Romania) were used as received. Polypropylene (PP; RS PRO Pad Spill Absorbent, RS Components Ltd., Corby, UK) sorbent pad was used as the industrial control, without any modification. Tap water was used without any additional treatment.

### 2.2. Solution Preparation

The solutions used to prepare the polymeric fiber mats were prepared with the aid of a magnetic stirrer (ARE; Fisher Scientific, Waltham, MA, USA) at 500 rpm under standard laboratory conditions. The polymer pellets required approximately 1 h of stirring to result in a homogenous, viscous, and hazy polymeric solution. We previously reported the optimization of centrifugal spinning conditions for SIBS [30]; thus, in this study, 25% *w*/*w* SIBS solution was prepared by dissolving SIBS pellets in a mixture of THF and toluene (80/20 mass ratio). Scouting experiments showed the optimal concentration to prepare PS fibers by centrifugal spinning to be 27% *w*/*w* PS solution prepared by dissolving PS pellets in a mixture of THF and DMF (25/75 mass ratio).

### 2.3. Centrifugal Spinning

**Figure 1:**
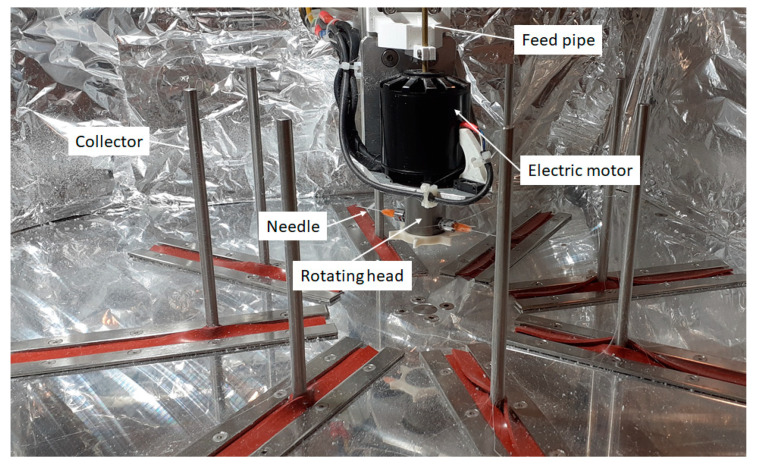
Main components of custom-built centrifugal spinning setup.

Fiber production by centrifugal spinning was performed on a custom-built setup [31]. Figure 1 shows the main components of the setup. The polymer solution is fed into the spinning head through PTFE tubing inserted into the feed pipe. The spinning head is equipped with two needles, which allow the polymer solution the burst out from the head during the spinning process and form the polymer solution jets. Fibers are collected on 8 radially positioned vertical rods.

The volumetric flow rate of the polymer solutions, provided by a syringe pump (KD Scientific, Holliston, MA, USA), was kept constant at 60 mL/h throughout all spinning experiments. The SIBS solution was spun using 25G needles at 8000 rpm, using a needle-to-collector distance of 75 mm. In case of PS, the conditions that resulted in the most consistent fiber generation included using 24G needles at 7000 rpm and applying a needle-to-collector distance of 100 mm. All spinning experiments were performed at ~22 °C and 40–60% relative humidity.

### 2.4. Microscopy

The morphology and fiber size were determined using images acquired by scanning electron microscopy (SEM). The PS and PP samples were investigated using a JSM-5200 (JEOL, Tokyo, Japan) scanning electron microscope with neat, not sputter-coated samples applying 25 kV as accelerating voltage. The SIBS samples were sputter-coated with Au (JFC-1200; JEOL, Tokyo, Japan), before SEM examination using a JSM-6380LA (JEOL, Tokyo, Japan) microscope at 5 kV acceleration voltage. Open access ImageJ software (version 1.52a, National Institutes of Health, Bethesda, MD, USA) was used to measure 100 fibers at different parts of the samples to determine the average fiber diameter and standard deviation with the equations of the normal distribution function.

Optical microscopy was used to study the morphology of the oil-containing samples. Imaging was realized using a Ceti Topic-T optical microscope (Medline Scientific, Chalgrove, UK) and an attached Canon DS126191 digital camera (Canon Inc., Tokyo, Japan).

### 2.5. Viscosity Measurement

The dynamic viscosity of the oils was measured using an IKA ROTAVISC Lo-Vi viscometer (IKA, Königswinter, Germany) at 22.5 ± 0.5 °C. Depending on the oil, different shear rates were used to stay within the range of the equipment. Based on the dimensions of the spindle and the oil container, the chosen 40, 50, 100, and 200 rpm rotational speeds resulted in shear rates of 2.89, 6.62, 7.25, and 14.5 1/s. The viscosity values were recorded 1 min after starting the rotation of the spindle.

### 2.6. Contact Angle Measurement (CA)

Videos at 240 fps of the water and oil droplets interacting with the fiber mats were taken using a Nikon SMZ645 (Nikon Corporation, Tokyo, Japan) optical microscope and a Xiaomi M2101K7BNY (Xiaomi Corporation, Beijing, China) smartphone camera. Images were extracted from the videos at specific time stamps. Water contact angles were measured 5 s after drop placement. The progression of the oil contact angle was monitored over time. For the sunflower and motor oils, the first measurement was taken at 0.05 s after impact, and the other four consecutive measurements were each executed at 0.1 s intervals. In the case of gear oil, the first measurement took place at 0.1 s, and the interval between the data points was 0.4 s, and with diesel, it was 0.025 s and 0.05 s, respectively. In each case, the CA was measured on 3 different droplets.

### 2.7. Oil Sorption Studies

The methods described in this section have ASTM F726-17 as their basis [6]; however, the indications were not followed entirely. The main difference is that the experiments presented in this paper are on a much smaller scale (200–300 mg specimens as opposed to >4 g given by the standard), and thus some parameters were altered to suit the circumstances. High-viscosity oils (1500–10,000 mPa·s) were left out, which was due to their unavailability. In addition, suggestions published by Bazargan et al. regarding the methodology were adopted [32].

**Figure 2:**
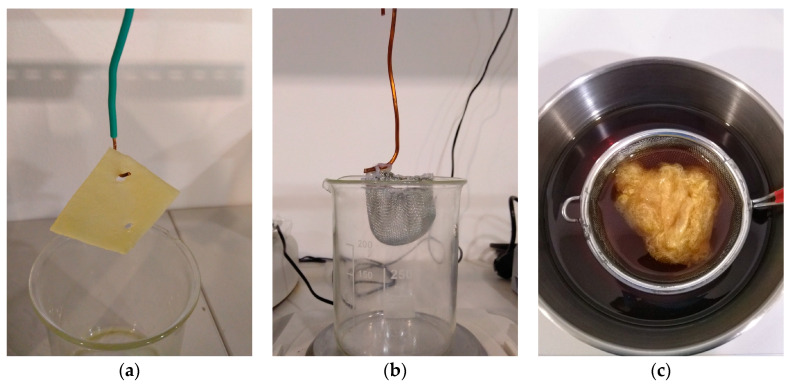
Devices for handling the specimens during oil sorption tests: (**a**) hook for the PP, (**b**) small basket for the SIBS, and (**c**) large basket for the PS.

Two separate experiments were performed in order to assess the oil sorption capabilities of the fiber mats:

1. Specimens of 300–400 mg (SIBS and control) and 200 mg (PS) were cut from the fiber mats. The reason for the difference in mass between the SIBS and PS samples was the difference in volume, as the average density of the PS fiber mat was lower, and the testing equipment would not have been able to properly contain a 300 mg PS specimen. In the contact phase, the SIBS and PS specimens were placed in a steel mesh basket and the basket was placed in a stainless steel vessel containing 200 mL of oil. The PP specimens were placed on the oil surface by themselves. The devices used to handle the specimens can be seen in Figure 2. The different meshes were necessary as neither the oil-soaked SIBS nor the PS specimens could be held by a hook. Since the PP samples maintained their structural integrity throughout the whole experiment, a simple hook was enough for handling. This, however, was not true for the SIBS and PS samples, and a supporting mesh was required. The SIBS specimens were contained in a smaller mesh basket, while the larger size of the PS specimens warranted the use of a larger basket, as shown in Figure 2.

The fibers were tested with various oils: sunflower oil, low-viscosity motor oil (5W30), high-viscosity gear oil (85W140), and diesel fuel. The specimens were exposed to the oils, and after 1, 3, 5, 10, 15, and 30 min, they were taken out and weighed. Each time, there was a 20 s drain period before weighing the samples, with the exception of the 30 min sampling. In this case of the last sampling, a 15 min release study (dripping phase) was conducted by hanging the specimens above a digital balance and recording the mass of the released oil at 20 s (reference 0), 1, 2, 3, 5, 10, and 15 min. The experiment was repeated 3 times for each polymer-oil combination.

The use of a mesh device can introduce several sources of error to the measurement. On the one hand, the sorption capacity is overestimated, as oil gets trapped within the mesh structure and between the mesh and the specimen, while it can also accumulate on top of the specimen much more easily, compared to the hook method. On the other hand, the oil retention—measured indirectly by the released oil mass—is artificially reduced, since some of the oil comes from the mesh itself or other sources. In order to account for these error sources, a compensation was performed. The compensated amounts were determined by a series of experiments, identical to the one already described, carried out with the PP control with all three devices.

2. Water (45 mL) and motor oil (1 g) were added to a beaker. The beaker was placed on a shaker (Grant-Bio PMS-1000i; Grant Instruments Ltd., Cambridge, UK) set to 260 cycles/min. Specimens of 150–200 mg (SIBS and PP control) and 100 mg (PS) were placed on top of the liquid surface. After 15 min, the shaker was stopped, and the system was allowed to sit for 2 min. Following that, the specimens were removed, drained for 1 min, and weighed. A second weight measurement was performed 24 h after the first one. The specimens were also tested with pure water, to obtain a baseline water uptake value. The steps were the same as with the oil–water mixture with an additional step to remove all of the water using paper towels and drying in open air for 24 h. Each measurement was performed with 3 specimens.

The oil sorption capacity (more precisely, mass specific sorption capacity) was calculated using the following equation:

(1) $$C_{sorption,m}=\frac{m_{specimen}-m_{mat}}{m_{mat}}$$

where *m_specimen_* refers to the mass of the oil-soaked fiber mat specimen at any given time, while *m_mat_* is the mass of the dry fiber mat specimen before oil sorption.

### 2.8. Porosity Calculations

The dimensions of the PP specimens were measured using a digital caliper and a screw-thread micrometer equipped with parallel plates, and the volume was calculated by assuming a rectangular prismatic shape. The volume of the SIBS and PS fiber mats was measured by gently placing the mats into 24 mL syringes with as little squishing as possible.

The density of the fiber mats was calculated by dividing the mass of the mat by its volume, as follows:

(2) $$\rho_{mat}=\frac{m_{mat}}{V_{mat}}$$

From here, the porosity could be calculated, as follows:

(3) $$porosity=1-\frac{\rho_{mat}}{\rho_{bulk}}$$

where *ρ_bulk_* is the density of the bulk polymer.

The reciprocal value of the fiber mat density is the mass-specific volume, given in cm^3^ fibers/1 g polymer, which is calculated as follows:

(4) $$V_{mat}^{spec,m}=\frac{V_{mat}}{m_{mat}}$$

The calculation of mass-specific surface area was performed based on the assumptions that the fibers were cylindrical and had a smooth surface. The surface area was calculated by multiplying the circumference of the fibers with their specific length, as follows:

(5) $$S_{fiber}^{spec,m}=\pi \cdot d_{fiber}\cdot L_{fiber}^{spec,m}=\pi \cdot d_{fiber}\cdot \frac{1}{\rho_{bulk}}\cdot \frac{4}{\pi \cdot {d_{fiber}}^{2}}$$

Whenever a mass-specific quantity (e.g., sorption capacity, surface area) was transformed into a volume-specific one, the original quantity was divided by the mass-specific volume of the fiber mat.

## 3. Results and Discussion

In order to have a basis of comparison, along with SIBS, two other materials were also tested. Melt-blown PP fibrous sheets are commercially available and are considered as a state-of-the-art device for oil collection. In addition, the chemical structure of PP is almost identical to that of PIB, so their affinity to oils should also be similar. The other chosen control was polystyrene, as SIBS itself also has 30% *w*/*w* of it, and other research groups have conducted substantial work with PS fibers.

### 3.1. Fiber Morphology

**Figure 3:**
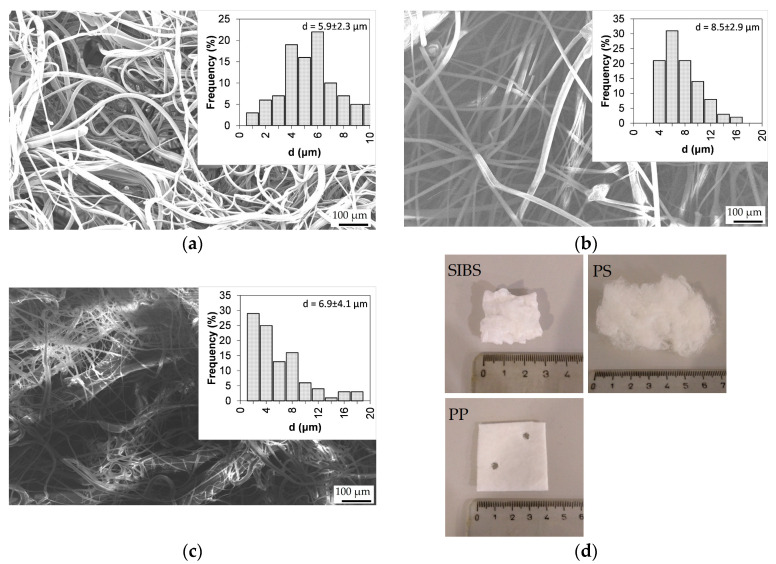
SEM micrographs of the (**a**) SIBS, (**b**) PS, and (**c**) PP fibers at ×100 magnification. (**d**) Physical appearance of the SIBS, PS, and PP fiber mats.

When the strands of centrifugally spun SIBS fibers were removed from the collector, they shrunk in length due to their elastic property and formed agglomerates. The PS fibers retained their formation during removal, and as such, a much looser structure was formed, similar to cotton wool. The appearance of the fiber mats is shown in Figure 3d, and it can be seen that 200 mg of PS fibers is significantly larger than 350 mg of SIBS fibers. The PP fiber mat was manufactured by pressing together multiple thinner fibrous layers to form a 2 mm thick sheet.

The SIBS fiber mat shown in Figure 3a consisted of randomly oriented fibers with an average diameter of 5.9 ± 2.3 μm. Many of the fibers were wavy or curled up, and the surface of the fibers appeared to be smooth.

The PS fibers (Figure 3b) were mostly straight and had an average fiber diameter of 8.5 ± 2.9 μm. There seemed to be some degree of surface roughness, although it is difficult to tell from the image. Other research groups acquired rough and porous fibers when spinning PS under similar conditions [19]. The fiber packing density is not representative, as the specimen required squishing during sample preparation.

The majority of the fibers making up the PP mat fell into the 2–6 μm range, but the presence of large (>10 μm) fibers pushed the average size above that of the SIBS fibers. The fibers were straighter than in case of SIBS, with some waviness present.

**Figure 4:**
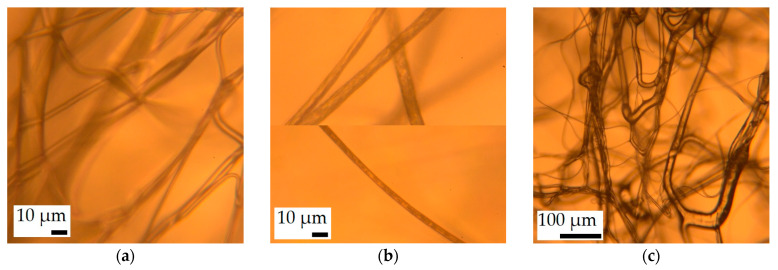
Optical micrographs of (**a**) SIBS and (**b**) PS fibers at ×400 magnification, and (**c**) PP fibers at ×100 magnification.

Images taken with the optical microscope gave some indication regarding the porosity of the PS fibers. While the SIBS and PP fibers were optically clear in the transmitted light (Figure 4a,c), the PS fibers seemed to be textured (Figure 4b), suggesting that the optical medium was non-continuous.

### 3.2. Oil Viscosity

Commercially available oils were chosen to cover a range of dynamic viscosity values. The viscosity data of the different oils can be seen in Table 1. Since the oils were dissimilar, there was not a single shear rate at which the viscosity of all of the oils could be measured.

The shear rates shown in the table were derived from rotational speeds of 40, 50, 100, and 200 rpm. It can be seen that gear oil with 725 mPa·s is about 6 times more viscous than the next most viscous oil, motor oil. However, it did not reach the viscosity of 1500 mPa·s recommended by the ASTM standard.

### 3.3. Contact Angles and Sorption Kinetics

The measurement of water and oil contact angles serves multiple purposes in the characterization of the fiber mats. First, it can show whether the fiber mat has water repelling properties, which is indicated by a large (>120°) WCA. It can also provide information about the speed of sorption into the fiber mat.

The average measured water contact angle was smallest for the SIBS mat, with 126.8 ± 6.4°, followed by the PS mat, with 131.5 ± 2.3°, and the highest was for the PP mat, with 134 ± 0.8°. The WCA of the PS mat was lower than the 142–148° reported for centrifugally spun PS mats by another group [19]. Overall, there was not a large difference, and all three fiber mats fell into the highly hydrophobic category, but none of them was superhydrophobic (WCA > 150°).

**Figure 5:**
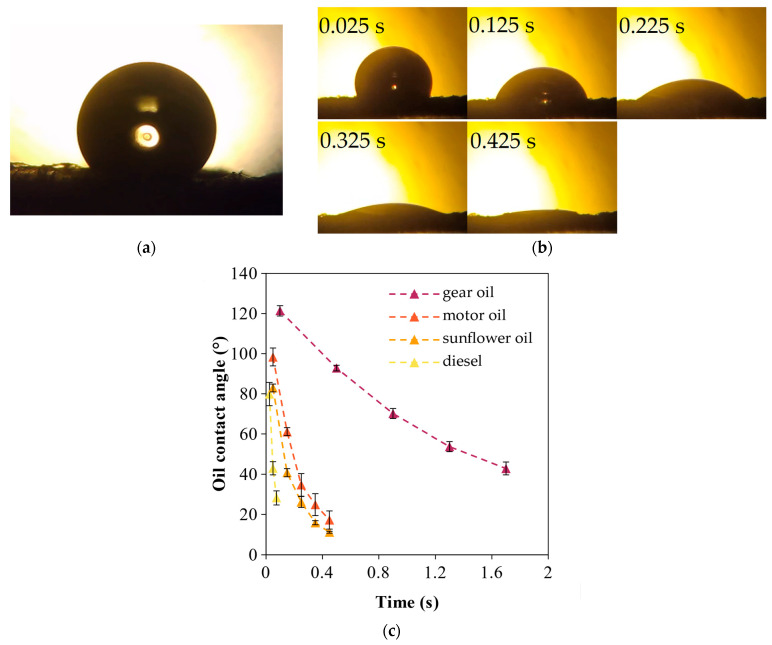
(**a**) Water droplet on an SIBS specimen with a CA of 131°. (**b**) Motor oil droplet on SIBS fiber mat at specific moments. (**c**) OCA progression over time on SIBS fiber mat for all oil types.

None of the mats were able to hold a stable OCA. While the sorption of the oil droplet into a fiber mat may seem instantaneous, there is an observable progression of the contact angle, as seen in Figure 5b. Initially (~0.02 s after drop placement) the oil droplet forms a well-pronounced CA, similar to water. In our study, the OCA correlated with the WCA, meaning that the higher the WCA, the higher the initial OCA for a given fiber mat. At this stage, the same principles that govern the formation of a WCA (described by the Wenzel and Cassie–Baxter models [33,34]) also apply to the oil droplet, so surface roughness and air pockets help facilitate this phenomenon. Shortly after that, the adhesive forces between the oil and the polymer take over and the oil starts spreading as well as penetrating into the fibrous structure.

The progression of the OCA for the SIBS mat can be seen in Figure 5c. Diesel had the fastest sorption and sunflower and motor oils had somewhat similar speeds, while it took the gear oil droplet almost 2 s to reach a state where there was not any visible CA. The sorption speed seemed to be correlated with the viscosity of the oil, with higher viscosity meaning slower sorption.

In order to compare the SIBS mat to the controls, the time it took for the droplet to penetrate into the fiber mat until no CA was detectable was measured (Table 2).

The results showed that the SIBS mat and PP control had mostly similar sorption times for the oils, with the exception of diesel, where a 3 times faster sorption was recorded for the PP mat. In general, the PS mat showed the shortest sorption times, with sunflower oil and diesel having half the speeds of those of the SIBS mat. The faster sorption kinetics in the case of PS could be explained with the loose structure of the PS samples, which allowed faster penetration into the porous structure, when compared to the denser samples of SIBS and PP.

### 3.4. Oil Sorption

The first of the two sorption studies aimed at measuring the maximum oil uptake of the fiber mats, as well as their oil retention capability. For this reason, enough oil was used to saturate the specimens, and there was no water involved so as to not introduce any additional uncertainty into the mass data. The experiment consisted of two parts. First the fiber mat was placed on top of the oil, and after a certain period of time, it was removed, drained, and weighed. This phase lasted 30 min (not counting the 20 s drain time and the time for weighing), and its purpose was to study how fast the fiber mats were saturated with oil. With the exception of the high-viscosity gear oil, 30 min was enough in all cases to reach saturation.

After the contact phase, the specimens were left to drain for 15 min, and the mass of the released oil was measured at certain intervals to determine the oil retention capabilities of the different materials. The 20 s drain time after each soaking period was initially established with sunflower and motor oils by doubling the time it took the oil dripping to reach less than 1 drop per second. While the chosen 20 s or 30 s specified by the ASTM 726 standard are arbitrary values, the desorption profiles show when the oil release slows down and eventually ceases. As such, it is a more reliable indicator of the sorption capacity.

**Figure 6:**
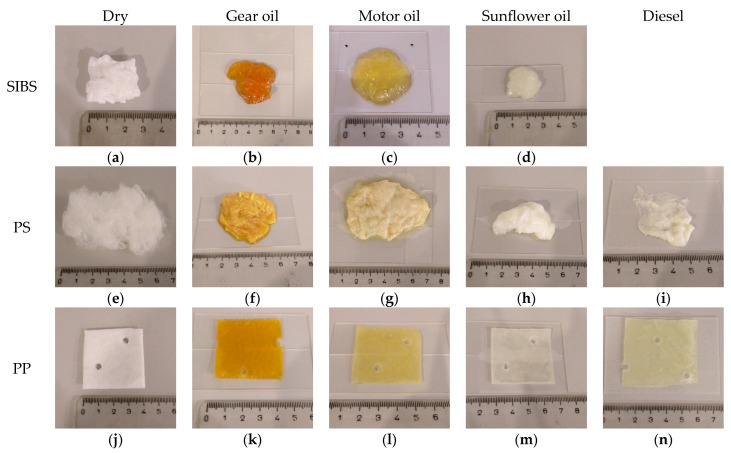
SIBS (**a**–**d**), PS (**e**–**i**), and PP (**j**–**n**) specimens before and after sorption. From left to right, dry, gear oil, motor oil, sunflower oil, and diesel specimens are presented. The SIBS has no diesel sample because it dissolved.

Figure 6 shows the specimens before and after the oil sorption experiments. Looking at the dry specimens, there is a clear difference in their appearance and size. Due to their shape, the dimensions of the specimens were difficult to quantify, so a ruler serves as a scale and thus the primary indicator. It can be seen that the PS specimen is twice as long as the SIBS or PP ones (the other dimensions are difficult to assess from the picture, but it was also wider and thicker), while having a lower mass (200 mg vs. ~350 mg). The PP mat, as a type I sorbent, has a much more compact structure, as multiple thin sheets are bound into a thicker one. At places, the sheets were melted together to achieve better structural stability. These spots were removed before the experiments because they added mass without the characteristic surface area of the fibers. The post-sorption specimens were also quite different from each other. The SIBS specimens turned into a semi-transparent gel-like substance and lost their original shape. There was no SIBS specimen to show paired with diesel, as it completely dissolved within the first minute of exposure. The PS samples, while still the largest, lost much of their volume. This was especially true with the lower viscosity sunflower oil and diesel, in which cases the fibrous structure collapsed considerably. The PP specimens looked the same both in shape and size as in their dry state, only their color was different.

**Figure 7:**
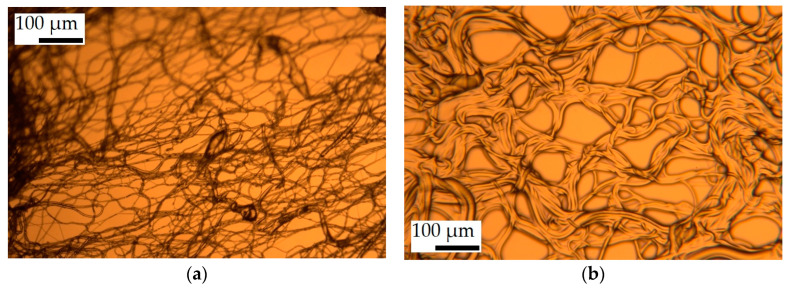

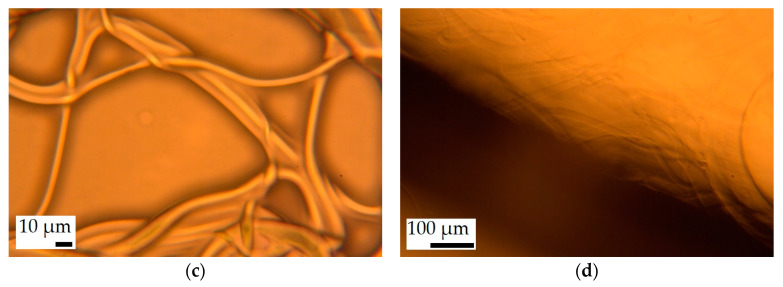
Optical micrographs of (**a**) dry SIBS fibers, SIBS fibers with a small amount of oil added at (**b**) ×100 and (**c**) ×400 magnification, and (**d**) a sample of a SIBS-motor oil specimen 4 weeks after the sorption experiment.

The interaction of oil with the SIBS fibers was also studied under optical microscopy. The specimen was slightly stretched before imaging in order to flatten it out, and for this reason, the fibers appear to be straighter compared to the SEM image. In Figure 7, (a) presents an image of the dry fibers, while (b) and (c) are images at different magnifications taken on the specimen 1 h after a trace amount of motor oil was added to it. The images show a thin oil layer enveloping the fibers, but there was not enough oil to fill the gaps.

Figure 7d presents a sample taken after the sorption test, the same one that is shown in Figure 7c. The sampling took place 4 weeks after the sorption experiment had concluded. While the specimen appeared gel-like to the naked eye and the specimen was saturated with motor oil, in the microscope image, the outlines of the individual fibers can still be seen. The fibers also appeared to be much thicker compared to the dry ones, indicating that over time swelling occurred, something that could not yet be observed after 1 h of oil exposure. This suggested that significant swelling did not occur during the sorption experiments either.

**Figure 8:**
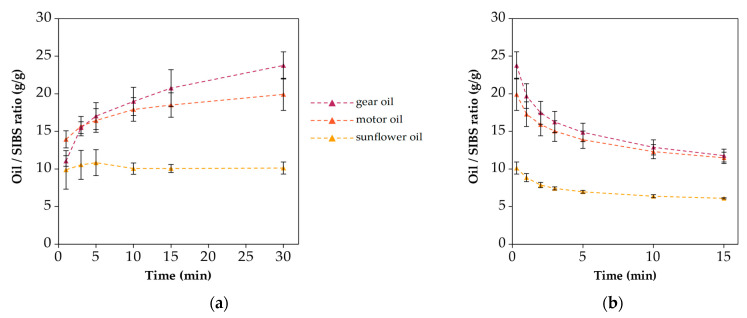
(**a**) Oil sorption and (**b**) desorption plots of the SIBS specimens.

Figure 8 presents the sorption and release behaviors of the SIBS specimens for the different oils.

The graphs only show three of the four oils, because the SIBS specimens dissolved in diesel. The specimens tested with sunflower oil became saturated by the 1 min mark, as indicated by the horizontal plot. However, motor and gear oils only reached 70% and 46% of the 30 min value, respectively. Even by 30 min, there was a slope present in the graphs of gear and motor oil, indicating that the specimens were not saturated yet. This trend can be explained with the sorption kinetics shown by the OCAs, and it can be attributed to the viscosity of the oils. The uptakes at 30 min were 10.1 ± 0.8 g/g for sunflower oil, 19.9 ± 2.1 g/g for motor oil, and 23.8 ± 1.8 g/g for gear oil. The maximum oil uptake does not carry too much importance if the specimens cannot contain that oil. After 15 min of dripping time, these values were reduced to 6.1 ± 0.1 g/g, 11.5 ± 0.7 g/g, and 11.8 ± 0.8 g/g respectively, losing between 40 and 50% of the oil. The desorption plots approached the horizontal line.

**Figure 9:**
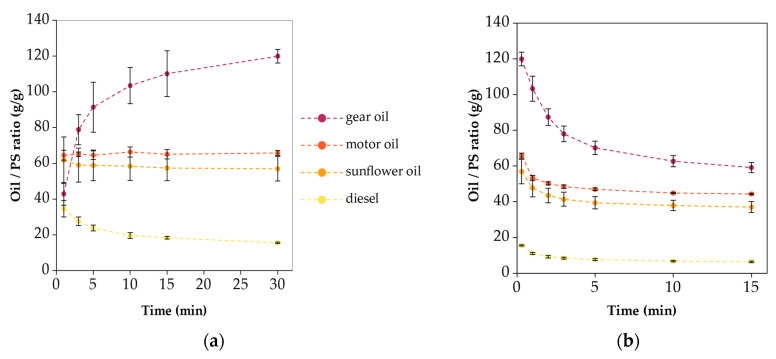
(**a**) Oil sorption and (**b**) desorption plots of the PS specimens.

Compared to the SIBS specimens, the PS specimens had much higher oil sorption values, peaking at 119.9 ± 3.85 g/g for gear oil, as can be seen in Figure 9a. As for the rest, 65.78 ± 1.38 g/g was recorded for motor oil and 56.9 ± 6.89 g/g for sunflower oil, whereas 15.5 ± 0.3 g/g was recorded for diesel. The gear oil plot had an increasing trend, indicating a slower uptake, and it did not completely reach saturation by 30 min. The sorption of motor and sunflower oils was fast enough that the first data point at 1 min already showed the maximum value. Interestingly, a decreasing trend was observed with diesel. This was an indication that some deterioration occurred in the fibrous structure. Based on the desorption plots, the motor oil specimens lost the least amount of oil in 15 min, 32.5%, while the diesel specimens lost the most, 58%.

**Figure 10:**
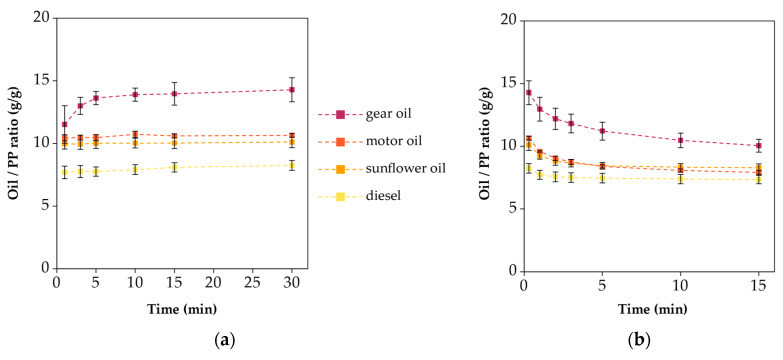
(**a**) Oil sorption and (**b**) desorption plots of the PP specimens.

The PP specimens performed the most consistently overall, although the oil sorption capacity was the lowest of the tested materials (Figure 10a). The sorption values at 30 min ranged from 8.24 to 14.28 g/g, and after the 15 min of dripping time, the gap decreased to only 2.7 g/g between the highest (gear oil) and lowest (diesel) values. Overall, the percentage of released oil was smaller than in the previous two cases, being 10–30%, which could be explained by its densely packed structure. The measured values were lower compared to the figures provided by the manufacturers, which were ~20 g/g.

**Figure 11:**
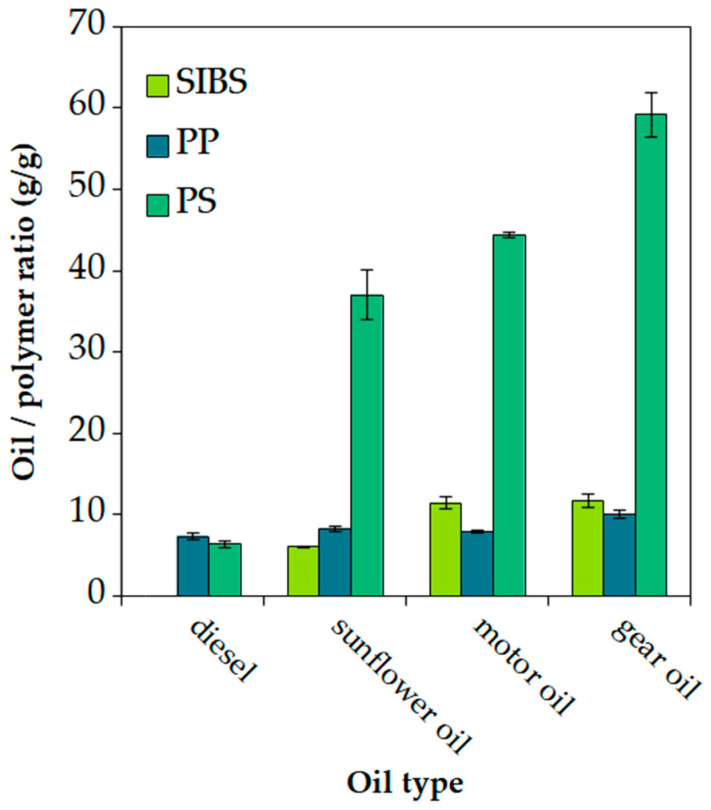
Oil retention values for each polymer–oil combination after 15 min desorption.

In general, we can state that the last data points of the release plots provided the most accurate measurements due to the fact that all of the transient phenomena ceased at this point. Thus, the error bars were the smallest for these data points. These data points are plotted in Figure 11. Based on the results, the SIBS fiber mat showed better sorption capacity than the industrial standard, PP, for both motor and gear oils; however, for the less viscous sunflower oil, it showed inferior sorption values. Both PP and SIBS were outperformed by PS.

For comparison, Table 3 contains some of the relevant sorption data from the literature for synthetic and natural fibers measured with similar methods. It should be noted that some of the conditions that strongly influence the sorption capacity (sample size, contact and drain times) vary significantly between the experiments. The materials presented are our control materials PS and PP, another styrenic triblock thermoplastic elastomer, styrene-ethylene-butylene-styrene (SEBS), PVC, as well as some natural fibers.

Doan et al. measured ~50 g/g sorption with oils with similar viscosity to sunflower and motor oils after 10 s of draining, which matches well with our results [19]. Others also measured similar sorption capacities with their less porous PS fibers [36,37]. By increasing the porosity of the fiber mats, the sorption capacity can be increased [35,36]. The table contains data acquired with melt-blown PP fiber mat specimens, and the oil sorption capacity of these is 3–4 times higher than that of commercial PP fiber mats, which is engineered into sheets. Zhu et al. used commercial PP sheets as the control, and their oil sorption values match with our measurement results [41].

In marine oil spills, a significant constituent of the contamination is crude oil. While this paper does not feature crude oil sorption with SIBS fibers, data from the literature can provide insight in this regard. There are multiple experiments in which, in addition to oils similar to the ones used in this paper, crude oil was also tested. The data shows that the sorption capacity of PVC fibers for crude oil is around half of that for motor oil [40], and diesel sorption is 10–20% lower than crude oil sorption for both PP fibers and porous butyl rubber sheets [22]. Combining this with the fact that diesel sorption is 3–10 times lower than motor oil or food-grade oils for PS fibers, it could be hypothesized that the crude oil sorption capacity of our SIBS specimens should be ~8 g/g after the 15 min drain period. This claim is further supported by experiments carried out with SEBS, a polymer similar to SIBS, in which 4.3 g/g crude oil uptake was measured after 30 s of exposure [42].

In general, the sorption capacity of natural fibers is lower than that of the synthetic fibers, with 6–20 g/g for cellulose and wool, ~40 g/g for the waxier kapok, and exceeding 80 g/g when surface modification was performed [26,27,28,29].

### 3.5. Oil–Water Separation

**Figure 12:**
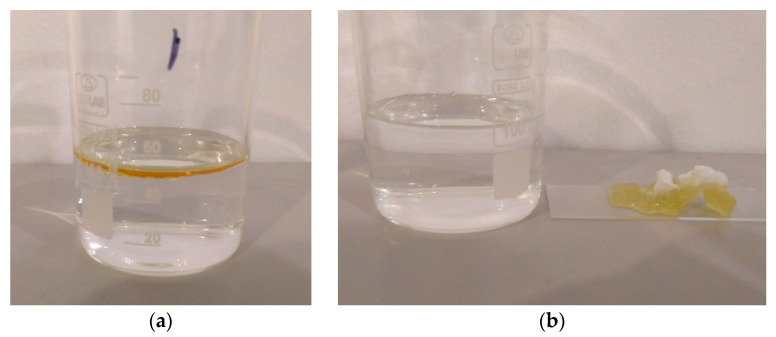
(**a**) Motor oil and water used in the separation study. (**b**) SIBS specimen with the removed oil.

While it is interesting to see the oil sorption capacity of the specimens in isolation, it is also important to study it in the context of the main area of application. For this reason, a layer of motor oil was poured on top of water, and the specimens were used to separate the oil from the water. The used quantity of 1 g oil was established so that it would not saturate any of the specimens (Figure 12a).

The ASTM standard recommends cyclic agitation of the container, specified at 150 cycles/minute with a 30 mm stroke distance [6]. Since the shaker used in this experiment had a stroke distance of about 10 mm, the frequency of agitation was increased to 260 cycles/minute in order to match the centripetal acceleration—assuming a circular motion—resulting from the conditions given by the standard. In addition, the specimens were also tested with water only to determine how much water they took up during the experiment.

Table 4 shows both the water and the total (oil + water) uptake after 15 min by the samples.

The water uptake shown in the table is attributed to small water droplets adhering to the surface of the fibers. The PS specimens showed higher water uptake values owing to the fact that they covered a larger area of the water surface due to their larger size. These droplets could be removed by shaking the specimens or with a paper towel, after which the specimens regained their original mass. When the specimens were exposed to the oil–water mixture, the total oil + water uptake exceeded the sum of the water and possible oil uptake for the individual samples. This means that the presence of the oil caused more water to adhere to the specimens. Assuming 100% oil sorption, there was 5.21 g/g water uptake for PS, 1.09 g/g for SIBS, and 2.71 g/g for PP. The excess water was released from the fiber mats over the course of 24 h, resulting in a total uptake virtually similar to that of the possible maximum oil uptake. The assumption that the specimens only lost water weight but not oil was supported by the fact that the surface on which the specimens rested was dry after 24 h. Since the specimens did not become saturated with oil, they all retained some of their original white color, as can be seen in Figure 12b. At the same time, the oil completely disappeared from the surface of the water.

It can be stated that all three materials were able to remove oil from water with almost 100% efficacy, and there was no significant difference in how they performed. All publications that contain an oil–water separation part show a 98–100% separation performance [28,36,38].

### 3.6. The Influence of Fiber Mat Porosity on the Oil Sorption Capacity

Table 5 contains the calculated values relating to the porosity of the specimens.

The density, porosity, and specific volume are all analogous quantities and are calculated from the same data. The bulk densities of *ρ*_SIBS_ = 0.95 g/cm^3^, *ρ*_PS_ = 1 g/cm^3^, and *ρ*_PP_ = 0.91 g/cm^3^ were used for the calculations. Based on our data points, a positive linear correlation can be seen between the specific volume and the oil sorption/retention capacity of the studied materials, meaning that the higher the specific volume of a fiber mat, the higher the oil sorption capacity. While the oil sorption of PS was 65.78 ± 1.38 g/g for motor oil and 119.9 ± 3.85 g/g for gear oil, with a specific volume of 85.02 cm^3^ fibers/g, SIBS with a roughly 6 times lower specific volume, 15.21 cm^3^ fibers/g, had uptakes of only 19.9 ± 2.1 g/g for motor oil and 23.8 ± 1.8 g/g for gear oil.

Taking the average fiber diameter into consideration, the specific surface area could be calculated. The results showed that (assuming a smooth fiber surface) while the mass-specific surface area of the PS mat was 66% of that of the SIBS mat, the volume-specific surface area was only 12%. The usual explanation for the high sorption capacity, other than the presence of voids between the fibers, is that the high mass-specific surface area of the porous fibers is responsible for high sorption capacity, since the oil has a lot of fiber surface to adhere to [19,35,36]. What this explanation does not emphasize is the importance of the overall dimensions (or specific volume/fluffiness) of a certain mass of fibers. Our results show that while the SIBS fibers had 700% more volume-specific surface area, than the PS fibers, the retained oil amount was only about 44% higher in the case of motor oil (0.754 g/cm^3^ for SIBS and 0.522 g/cm^3^ for PS) and 11% higher in case of gear oil (0.773 g/cm^3^ for SIBS and 0.695 g/cm^3^ for PS). This suggests that increasing the specific volume (either by increasing the fiber porosity or reducing the fiber packing density) is just as important as achieving a high specific surface area. That being said, fiber porosity and surface area are correlated, and a higher fiber porosity increases the specific volume of the fiber mats, thus more fibers are created from the same amount of polymer solution.

The hypothesis is that while adhesion to the fiber surface is necessary to keep the oil in place, the overall volume of the fiber mat and the associated voids between the fibers play a more significant role in containing large amounts of oil.

### 3.7. Reusability

The attempt to remove the oil from the SIBS and PS fiber mats by pressing resulted in the destruction of the fibrous structure. The SIBS mat became an oil-soaked sheet and lost its oil sorption capacity. The PS fiber mat broke into tiny pieces under the mechanical load, most probably due to the rigid nature of the material. In both cases, about 50–60% of the oil remained in the mats. The PP mat, on the other hand, released 80% of the collected oil, mostly regained its original white color, and seemed undamaged.

## 4. Conclusions

In conclusion, bead-free SIBS and PS fiber mats were produced with centrifugal spinning. The oil sorption kinetics of the produced SIBS fiber mats were investigated by contact angle measurements, indicating a strong correlation between the oil viscosity and sorption kinetics, which was further supported by the oil sorption experiments. The thermoplastic elastomer SIBS fiber mat proved to be a promising device for oil sorption and separation. The results indicate that SIBS outperformed the industrial standard PP in oil sorption for motor and gear oils. The PS fiber mat with higher specific volume outperformed both the SIBS and PP fiber mats in the oil sorption and retention tests. Oil–water separation tests indicate that SIBS performed similarly to PP and PS, removing ~100% of the oil.

Our results indicate that oil sorption capacity is influenced not only by the specific surface area but also by the specific volume of the prepared fiber mats. This hypothesis poses an interesting engineering challenge to prepare SIBS fiber mats with a higher specific volume to potentially increase their sorption capacity.

## Figures and Tables

**Table 1:** Dynamic viscosity values of the oils at various shear rates at 23 °C.

Shear Rate (1/s)	2.89	3.62	7.25	14.5
Oil Type	Dynamic Viscosity (mPa·s)			
Gear oil	725	-	-	-
Motor oil	-	126.6	128.7	-
Sunflower oil	-	-	57.6	66
Diesel	-	-	8.6	10.3

**Table 2:** Comparison of the average sorption time of a single droplet for each polymer and oil type.

	Droplet Average Sorption Time (s)			
Specimen	Gear Oil	Motor Oil	Sunflower Oil	Diesel
SIBS	3.96 ± 1.75	0.64 ± 0.07	0.58 ± 0.15	0.21 ± 0.09
PS	2.11 ± 0.48	0.48 ± 0.1	0.27 ± 0.12	0.11 ± 0.05
PP	4.24 ± 0.41	0.62 ± 0.09	0.42 ± 0.08	0.08 ± 0.03

**Table 3:** Oil sorption data of synthetic and natural fibrous structures.

Material	Oil Type	Sorption Capacity (g/g)	Sample Size (mg)	Contact Time (s)	Drain Time (s)	Reference
PS	silicone oil pump oil vegetable oil	32–47 34–48 28–45	10	3600	10	[19]
PS	silicone oil pump oil sunflower oil diesel	350–840 210–650 100–450 30–60	10	3600	60	[35]
PS	silicone oil motor oil peanut oil diesel	20–80 40–130 40–112 3–7	1000	2400	300	[36]
PS	vegetable oil	55–124	25	60	30	[37]
PS (recycled EPS)	motor oil	15	120	20	n/a	[38]
PP	motor oil peanut oil	38–65 25–52	100	3600	1800	[21]
PP	diesel crude oil	11.4 15.5	2000	300	n/a	[22]
PP	silicone oil machine oil soybean oil	35 45 32	n/a	n/a	30	[39]
PVC	motor oil hydraulic oil crude oil	35 24 16–19	250	3600	120	[40]
PVC/PS	motor oil peanut oil diesel	146 119 38	1000	3600	120	[41]
SEBS	transformer oil diesel crude oil	4.8 dissolved 4.3	n/a	30	n/a	[42]
butyl rubber porous sheet (non-fibrous)	diesel crude oil	20.3 23	2000	n/a	30	[22]
PLA	silicone oil pump oil sunflower oil	30 28 25	8	n/a	30	[20]
Natural fibers						
cellulose/lignin (jute)	motor oil 5w30 diesel crude oil	7 7 6	n/a	900	1800	[26]
sheep wool	motor oil 5w30 motor oil 15w40	9 11	n/a	900	1800	[27]
modified kapok	motor oil diesel	86.5 60.6	0.1	1800	30	[28]
kapok/cotton	engine oil vegetable oil diesel	45 44 40	ASTM F716			[29]
cotton	engine oil vegetable oil diesel	21 20 16.5	ASTM F716			[29]

**Table 4:** Liquid uptake values of the SIBS, PS, and PP specimens during the oil–water separation study, and 24 h after that.

Specimen	Measured Water Uptake (g/g)	Possible Maximum Oil Uptake (g/g)	Measured Oil + Water Uptake at 1 min (g/g)	Measured Oil + Water Uptake at 24 h (g/g)
SIBS	0.4 ± 0.07	4.90	6.00 ± 0.36	5.02 ± 0.37
PS	3.26 ± 0.5	10.67	15.89 ± 1.55	10.55 ± 0.58
PP	0.2 ± 0.1	4.96	7.67 ± 0.4	5.23 ± 0.1

**Table 5:** Porosity data of the SIBS, PS, and PP fiber mats.

Specimen	Density (10^−2^ g/cm^3^)	Specific Volume (cm^3^/g)	Porosity	Average Fiber Diameter (μm)	Mass Specific Surface Area (m^2^/g)	Volume Specific Surface Area (m^2^/cm^3^)	Motor Oil Retention (g/cm^3^)	Gear Oil Retention (g/cm^3^)
SIBS	6.57 ± 0.21	15.21	0.931	5.9	0.71	0.0467	0.754	0.773
PS	1.17 ± 0.34	85.02	0.988	8.5	0.47	0.0055	0.522	0.695
PP	7.73 ± 0.28	12.92	0.915	6.9	0.637	0.0493	0.612	0.777

## Data Availability

Data are contained within the article.
